# Effect of Three Different *Aloe vera* Gel-Based Edible Coatings on the Quality of Fresh-Cut “Hayward” Kiwifruits

**DOI:** 10.3390/foods9070939

**Published:** 2020-07-16

**Authors:** Roberta Passafiume, Raimondo Gaglio, Giuseppe Sortino, Vittorio Farina

**Affiliations:** Department of Agricultural, Food and Forest Sciences (SAAF), Università degli Studi di Palermo, Viale delle Scienze, 90128 Palermo, Italy; roberta.passafiume@unipa.it (R.P.); raimondo.gaglio@unipa.it (R.G.); vittorio.farina@unipa.it (V.F.)

**Keywords:** post-harvest technologies, sustainability, hydroxypropyl methylcellulose, lemon essential oil, natural antimicrobial agents, *Actinidia deliciosa* A

## Abstract

In recent years, the market for minimally processed fruit has increased. Fresh-cut fruits are characterized by a short shelf life due to the processing phases that accelerate the ripening courses. The aim of this work is to analyze the effect on the quality of fresh-cut Hayward kiwis of three different edible coatings based on (1) *Aloe vera* gel, (2) *Aloe vera* gel + hydroxypropyl methylcellulose and (3) *Aloe vera* gel + lemon essential oil. Fruit firmness, weight loss, color, soluble solids content, titratable acidity, microbial load and sensory analysis were evaluated as fresh after 2, 4, 7 and 10 days. *Aloe vera* gel and *Aloe vera* gel + lemon essential oil maintained the best values, as they acted as a barrier to gas exchange and further reduced the microbial load. These results were confirmed by sensory analysis: *Aloe vera* gel + hydroxypropyl methylcellulose does not alter the natural taste of kiwi slices, *Aloe vera* gel + lemon essential oil gives the characteristic taste of lemon essential oil and *Aloe vera* gel gives an herbaceous taste. The *Aloe vera* gel, in combination with these additives, maintains the ability to preserve the quality of fresh-cut kiwifruit.

## 1. Introduction

Ready-to-eat food products are the most requested on the market in recent years. The market for fresh-cut fruit in Europe began in the early 1980s and has been characterized by a double-digit growth in the last decade. These products are characterized for their convenience, but, on the other hand, they have a very short shelf life due to the damage of peeling, cutting and storage. These processes alter the physicochemical characteristics of the fruit [1,2], with a consequent acceleration of ripening. The search for methods that aim to delay these negative effects is of great interest to all the stakeholders involved in the production and distribution of fresh-cut fruit. One of the fruits affected by the rapid decay is “Hayward” kiwifruit (*Actinidia deliciosa* A. Chev.) due to its sensitivity to ethylene and respiration rate [3]. Kiwifruit is a climacteric berry appreciated among consumers due to its high nutritional value [4]. In the 1930s, the Hayward variety was selected, and during the 1960s, the marketing of kiwis from New Zealand began to grow, and they acquired the name “kiwifruit”, referring to the origin of the exports. Afterward, the fruit became popular due to its health attributes (rich in vitamin C, potassium, fibers and low-calorie contents) [5]. In Europe, Italy is the country with the world’s largest surface area under cultivation. Many preservation technologies have been used to prolong the post-harvest quality of whole kiwis [3,6,7,8,9,10,11,12]. However, there are few studies concerning the post-harvest preservation of ready-to-eat “Hayward” kiwifruit; most of them concern the use of conventional techniques such as dipping [13,14,15]. The growing popularity of high-quality food with good nutritional properties is leading to increased interest in totally natural preservation techniques, considering the recent consumer and market interests in sustainability, starting from cultivation techniques and their impact on the environment [16] to the fruit production supply chain [17]. In this context, natural-based edible coatings seem to maintain unaltered the organoleptic characteristics of the fruit and to satisfy producers and consumers, without the addition of chemicals that could be harmful to humans and the environment. Several compounds have been used as coating materials on fruits, including alginate, cellulose, lipids, chitosan, mucilage, starch, wax and zein [18,19]. Concerning kiwifruit, pullulan, Semperfresh^TM^, calcium caseinate, chitosan and lipid and protein-based solutions were evaluated as edible coatings [3,7,20]. These matrices delay the softening and browning of the pulp, inhibiting water loss and gas exchange, and can prolong post-harvest life compared to uncoated fruit due to their antioxidant and antimicrobial activity [21,22,23].

Currently, one of the most studied natural matrices of edible coatings is the gel extracted from *Aloe vera* leaves; its gel is rich in bioactive compounds that have been widely used for their medicinal and therapeutic properties [24], including anthraquinones; aloin α and β; hemodin; aloe-mannan (polysaccharide); lecithin; almost all essential amino acids (20 out of 22); many vitamins (A, B1, B2, B6, B12, C and E); mineral salts (zinc, magnesium, manganese, selenium, calcium and iron); acetylsalicylic acid and choline amylase. As a coating, it has been extensively used in concentrations between 50% and 100% in products such as nectarines [25], mangoes [26], apples [27,28], papayas [29] and peaches [30] in order to reduce respiration rates, ethylene production, weight loss and microbial load due to its antibacterial and antifungal activity [31,32]. However, as demonstrated by Farina et al. [27,29], *Aloe vera* gel-based edible coatings without the addition of additives (such as polysaccharides or lipids) show no improvement in fresh-cut fruits compared to other edible coatings added with these natural additives. An example is hydroxypropyl methylcellulose, one of the edible cellulose derivatives that are still little used in the formulation of edible coatings [33]. This element of natural origin is readily available, transparent, odorless, colorless, oil-resistant and water-soluble. It is also nontoxic and chemically stable [34]. Its safety in food use has been confirmed by the Joint FAO/WHO Expert Committee on Food Additives and Contaminants (JECFA) [35]. Recent studies have shown that edible coatings based on hydroxypropyl methylcellulose (HPMC) delay the loss of flesh color, weight and firmness in fresh-cut apples [27]. The important additives of edible coatings are the fungicide and antioxidant agents. For this purpose, essential oils have been extensively tested [36,37,38] in several fruits, but there are no studies concerning the application of lemon essential oil on fresh-cut kiwifruit.

Essential oils are natural substances extracted from fruit and vegetables. They are designated as Generally Regarded as Safe (GRAS) by the United State Food and Drug Administration (FDA). Lemon essential oil represents the largest share of extraction in the world and is extracted from the genus *Citrus* (L. 1753), which includes 16 species [39]. In fact, *Citrus* essential oils contain volatile constituents, such as limonene, aldehydes (citral), esters, alcohols (linalool), acids and many others [40]. These constituents have antifungal and antioxidant properties and can be used as alternatives to chemical additives in edible coatings [41]. Its potential toxicity is achieved by incorporating very low doses into the formulation of edible coatings [42].

On the basis of our previous study [27], this work aims to provide evidence of how the edible coating already used on fresh-cut apples, composed of *Aloe vera* gel, hydroxypropyl methylcellulose and lemon essential oil, is useful to improve even the organoleptic characteristics of fresh-cut kiwifruit during the storage period by monitoring the physicochemical parameters, microbial load and by evaluating the sensory quality.

## 2. Materials and Methods

### 2.1. Vegetal Material

In November 2019, one hundred and fifty “Hayward” kiwifruits were harvested in Torrenova (ME), Sicily (Southern Italy) at commercial ripening. They were selected for regular shape, uniform size, absence of visible defects and soluble solid contents (SSC) of 13 °Brix. Fruits were transported to the laboratories of the Department of Agricultural, Food and Forestry Sciences of the University of Palermo, where they stored for three days before being processed.

### 2.2. Preparation of Aloe vera Gel

One kilogram of mature leaves of *Aloe vera* were harvested at the experimental field of the University of Palermo, washed in tap water and immersed in 100 µL·L^−1^ of sodium hypochlorite for 5 min. The gelatinous parenchyma was separated from the leaves by means of a stainless-steel knife removing the external epidermis. It was triturated using an ultra-Turrax T25 (Janke and Kunkle, IKa Labortechnik, Breisgau, Germany) for 5 min at 24,500 rpm to form a homogeneous substance and filtered to remove the fibrous portion. Five-hundred milliliters of extract were obtained.

Based on our previous studies [14,27], the percentage of *Aloe vera* gel was established at 40% of its volume in 300 mL of water (*v/w*) for sensory acceptability due to a bitter taste that occurred at higher concentrations.

### 2.3. Coating Formulation

The edible coatings have been made with products of exclusively natural origins. *Aloe vera* gel is the basis of all treatments, except for the control sample. Hydroxypropyl methylcellulose (HPMC) and lemon essential oil are used in implementing the antifungal and gelling properties of the coatings.

Hence, three different edible coatings were tested in this work and compared with the untreated sample, according to Farina et al. [27]:

- AVG, for samples treated with 40% *v/w* of *Aloe vera* gel only,
- HPMC, for samples treated with 40% *v/w* of *Aloe vera* gel and 0.1% *v/w* of hydroxypropyl methylcellulose,
- LEO, for samples treated with 40% *v/w* of *Aloe vera* gel and 1% *v/w* of lemon essential oil and
- CTR, for the untreated samples (treated only with chlorinated water 0.5% *v/w*).

The solutions were kept at 40 °C for 90 min into an autoclave and centrifuged at 3000 rpm for 20 min by means of ultra-Turrax.

### 2.4. Sample Preparation and Experimental Design

The working area and all the utensils and surfaces in contact with the fruit during processing were washed and sanitized with 200 µL·L^−1^ of sodium hypochlorite solution to have a maximum sanitizing effect.

Kiwifruits were washed in tap water and then immersed in chlorinated water (0.5% *v/w*) for 10 min. Next, the whole fruits were divided into four lots (number of treatments). Afterwards, the kiwis were peeled and cut with a sterilized stainless-steel knife into 1.5-cm round slices.

The applications of edible coatings (for the treatments) and the chlorinated water (for the CTR) were performed through a spraying technique, with an airbrush (0.8-mm nozzle) powered by N_2_ for 2 min. Next, all the sliced kiwis were air-dried for about 30 min.

Sixty PET (polietilentereftalato) trays were equipped. In particular, three slices per tray were stored at 4 ± 1 °C and 90% ± 5% relative humidity (RH) in dark conditions. Each tray (125 mm × 115 mm and 150 cc) was thermally sealed on the top with a bioriented polypropylene (BOPP) film of 50 mm of thickness. The permeability of the BOPP was 2.004 mL O_2_ m^−2^ d^−1^ atm^−1^ and 3.824 mL CO_2_ m^−2^ d^−1^ atm^−1^. The physicochemical, microbiological and sensory analyses were carried out in three replicates and on day 0 (as fresh), day 2, day 4, day 7 and day 10. Then, 180 kiwifruit slices (collected from 150 fresh kiwis) were sampled and used as follows: 3 slices × 3 replicates × 4 treatments (CTR, AVG, HPMC and LEO) × 5 times of storage (0, 2, 4, 7 and 12 d). Therefore, a total of forty-five slices of kiwi were stored for each treatment.

### 2.5. Physicochemical Analysis

The weight loss of each tray was measured throughout the storage period by a two-decimal precision digital scale (Gibertini, Milan Italy), and the values were expressed as relative percentages of the mean and standard deviation (1):

Weight loss (%) = [(Wi − Wd)/Wi] × 100(1)

where Wi is the initial weight, and Wd is the weight measured during storage.

A Minolta colorimeter (Chroma Meter CR-400, Konica Minolta Sensing Inc., Tokyo, Japan) was used to evaluate the color change in the circumference of the kiwifruit slices, in order not to influence the data with the presence of seeds and columella. First of all, the instrument was calibrated using a standard white plate. The color space was shown as follows: brightness (L* value) from 0 (black) to 100 (white), greenness (a* value) from −a (greenness) to +a (redness) and yellowness (b* value) from −b (intensity of blue color) to +b (intensity of yellow color) [43]. From the obtained value of L*, a* and b*, the total color difference was calculated following the formula: ∆E* = [(L2* − L1*)^2^ + (a2* − a1*)^2^ + (b2* − b1*)^2^]^1/2^, and then, a color table was created by converting the CIEL*a*b* color space into the red/green/blue (RGB) scale.

The browning of the kiwi slices was carried out following Formula (2) of Ruangchakpet and Sajjaanantakul [44]:

Browning Index (BI) = [100 (x − 0.31)]/0.17](2)

where x = (a* + 1.75L*)/(5.645L* + a* − 0.3012b*).

A TA.XTPlus Texture Analyzer (Stable Microsystems, Ltd., Godalming, UK) connected to a personal computer equipped with a 50-N load cell and at room temperature was used to analyze the firmness. The following conditions were set according to the instrument manufacturer recommendations: pretest speed 5 mm/s, test speed 1 mm/s, post-test speed 5 mm/s, penetration distance 4 mm into the fruit and trigger force 5 *g*. A 4-mm diameter (P/4) stainless-steel flat probe was used to assess the texture. The force-distance curves were obtained from the puncture tests, and the hardness was taken as the area under the curve expressed in N·mm^−1^.

The juices of the kiwifruit slices were extracted by a squeezing–centrifugal machine (Ariete, De’Longhi Appliances s.r.l—Italian small appliance, Firenze, Italy) and was determined the soluble solids content (SSC) through a digital refractometer ATAGO (Atago Co, Ltd., Tokyo, Japan) at 20 °C and expressed as °Brix and the titratable acidity (TA) by titration with 0.1-N NaOH up to pH 8.1 with a Crison Compact titrator pH meter (Crison Instruments, SA, Barcelona, Spain) and expressed as g of citric acid per L^−1^. Contemporarily the pH of the juice was recorded, and, finally, the ratio between the SSC and TA was also calculated.

### 2.6. Microbiological Analysis

Fruit samples and edible coatings were microbiologically investigated in order to investigate on their quality, hygiene and safety aspects. Cell suspensions of edible coating samples were subjected to decimal serial dilutions in Ringer’s solution (Sigma-Aldrich, Milan, Italy), while fruit samples (25 g) were first homogenized in 225 mL of Ringer’s solution (Sigma-Aldrich) by the Bag-Mixer 400 stomacher (Interscience, Saint Nom, France) for 2 min at the highest speed (blending power 4) and then serially diluted. Different microbial groups were investigated as follows: total mesophilic microorganisms (TMM) on plate count agar (PCA), incubated at 30 °C for 72 h, total psychrotrophic microorganisms (TPM) on PCA, incubated at 7 °C for 7 d, pseudomonads on pseudomonas agar base (PAB) added with cetrimide Fucidin cephaloridine (CFC) supplement, incubated at 25 °C for 48 h, members of the Enterobacteriaceae family on violet red bile glucose agar (VRBGA), incubated at 37 °C for 24 h and yeasts and molds on Yeast extract Peptone Dextrose (YPD) agar supplemented with 0.1-g/L chloramphenicol to avoid bacterial growth, incubated at 25 °C for 48 h and 7 d, respectively. Plate counts were performed by the spread plate method [45] inoculating 100 µL from each diluted cell suspension of edible coatings and fruit samples. All media and supplements were purchased from Oxoid (Milan, Italy). Microbiological counts were carried out in triplicate at each collection time.

### 2.7. Sensory Analysis

Sensory analysis was performed on a sample of slices after 0, 2, 4, 7 and 10 days of cold storage. The sensory analysis was conducted at the postharvest laboratory of the University of Palermo in November 2019. According to Farina et al. [46], the sensory evaluation test was performed by an evaluation team consisting of 10 panelists (25–50 years of age and 70% males and 30% females) with good backgrounds and knowledge of the details of this evaluation. All panelists were trained and had broad expertise in the sensory evaluation of fruits.

The ten panelists evaluated the marketability and physiological alterations, as well as the total liking, according to Benìtez et al. [14]:

- marketability parameters, i.e., visual appearance (VA), color (C), sweetness (S), aroma intensity (AI), acidity (A) and fibrous meat texture (FFT) and
- parameters of physiological alterations, i.e., astringency (As), juiciness (J), dehydration (D) and rare flavors (RF).

The last descriptor tries to give an overall assessment (OA) of freshly cut fruit.

For the marketability parameters, the judges evaluated the descriptors as follows: 1 = extremely unacceptable, 2 = unacceptable, 3 = limit of marketability, 4 = good and 5 = excellent. For the physiological alteration parameters, the judges assessed the descriptors as follows: 1 = extreme presence, 2 = important presence, 3 = acceptable, limit of consumption, 4 = light presence and 5 = absence.

The parameters were evaluated separately, because they have opposite evaluation indices: the marketability parameters indicate the limit of marketability beyond which, according to the judges, a product is no longer saleable; the physiological alteration parameters indicate the limit beyond which, according to the judges, a product is no longer edible, because it is completely degraded.

Concerning the rare flavors, the judges identified the type of rare flavors that they could detect. In fact, the panel test was carried out according to Marsh et al. [47], which associates flavors with aroma compounds. In particular, a grass odor corresponds to cis-3-hexenel 40 µL·L^−1^, a sulfur odor corresponds to a cooked egg flavor, a vomit odor corresponds to 50 g·L^−1^ of butyric acid and fruit candy corresponds to ethyl-hexanoate 700 µL·L^−1^, while acidity corresponds to 1 g·L^−1^ of malic acid, sweetness to 20 g·L^−1^ of sucrose and fibrous flesh texture to a canned pineapple flavor.

### 2.8. Statistical Analysis

All data were tested for differences between treatments and sampling times using the two-factor analysis of variance (ANOVA; general linear model) followed by Tukey’s multiple range test for *p* ≤ 0.05, except for the microbiological analysis, where a *p*-value ≤ 0.001 was considered significant. All statistical analyses was conducted using Statgraphics Plus software (vs. 5.1, Statpoint Technologies Inc., Warrenton, VA, USA) and XLSTAT software version 9.0 (Addinsoft, Paris, France), both providing the same results.

## 3. Results and Discussions

### 3.1. Flesh Color and Browning Determination

**Figure 1:**
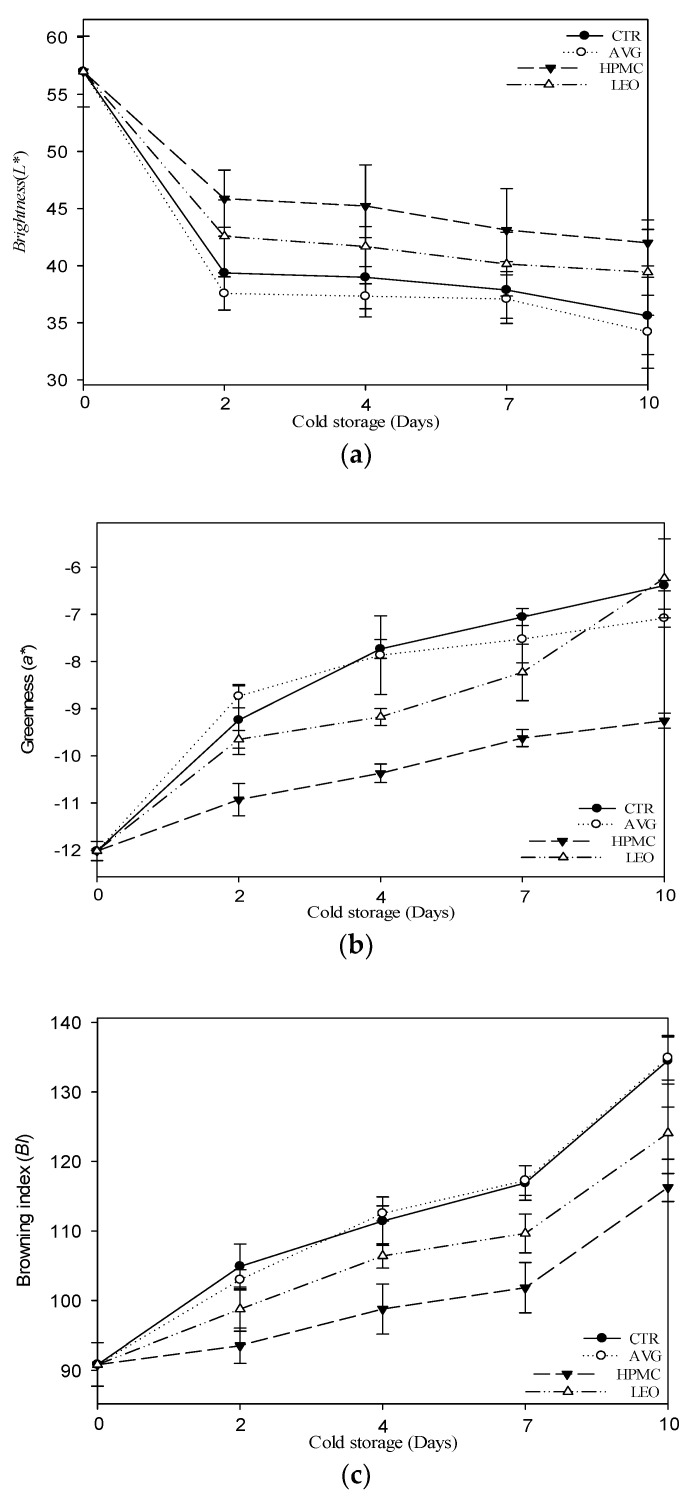
(**a**–**c**) Brightness (L*), greenness (a*) and browning index (*BI*) of kiwifruit slices treated with AVG, HPMC and LEO coatings and not treated (CTR) at day 0 and after 2, 4, 7 and 10 days of cold storage at 4 ± 1 °C and 90% ± 5% relative humidity (RH) in dark conditions. Values indicate the means, and the bars indicate the standard deviations of the replicates (*n* = 3). Abbreviations: CTR, uncoated fruit slices, AVG, fruit slices coated with 40% *v/w* of *Aloe vera* gel only, HPMC, fruit slices coated with 40% *v/w* of *Aloe vera* gel + 0.1% *v/w* of hydroxypropyl methylcellulose and LEO, fruit slices coated with 40% *v/w* of *Aloe vera* gel + 0.1% *v/w* of lemon essential oil.

Consumers a choose fresh-cut fruit by primarily considering its aspect and color. In all our samples, the brightness of the flesh (L*) decreased during the storage time, but the results were affected by the use of edible coatings. Considering the initial L* values of the fresh and untreated kiwi slices, after two days of storage, all the treatments suffered a rapid decline in this value, probably due to the low storage temperatures (Figure 1a). However, the HPMC and LEO treatments resulted in fewer changes in brightness than the CTR and AVG, which showed similar behaviors throughout the storage times and became browner than the others. Similar results were obtained for apple slices from Farina et al. [27] and Chauhan et al. [48], which showed that the samples treated with *Aloe vera* gel only were lower than the other treatments, while maintaining high-quality apple slices compared to the control. As regards results specifically on kiwifruits, Agar et al. [1] reported a similar reduction in brightness when the kiwi slices were stored with MAP (modified atmosphere packaging) technology.

**Figure 2:**
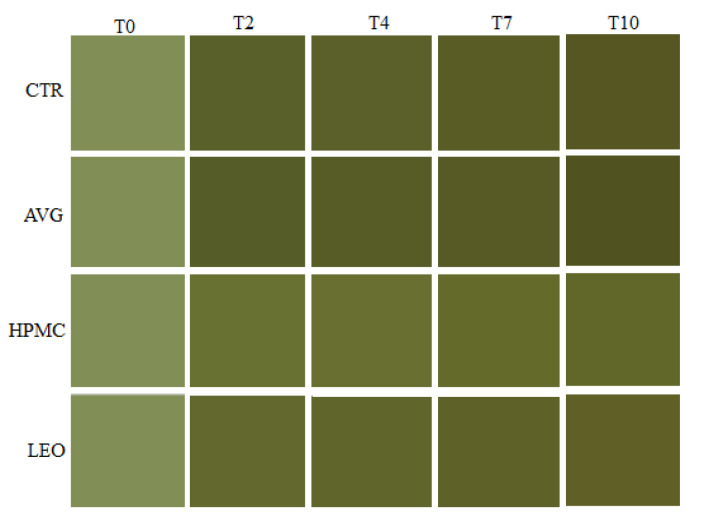
Color table obtained from the results of the total color difference (∆*E*) recorded in the CIEL*a*b* color space and converted to the red/green/blue (RGB) scale through the www.e-paint.co website. This figure shows the differences between the coated (AVG, HPMC and LEO) and uncoated (CTR) kiwifruit slices at day 0 and after 2, 4, 7 and 10 days of cold storage at 4 ± 1 °C and 90% ± 5% relative humidity (RH) in dark conditions. Abbreviations: CTR, uncoated fruit slices, AVG, fruit slices coated with 40% *v/w* of *Aloe vera* gel only, HPMC, fruit slices coated with 40% *v/w* of *Aloe vera* gel + 0.1% *v/w* of hydroxypropyl methylcellulose and LEO, fruit slices coated with 40% *v/w* of *Aloe vera* gel + 0.1% *v/w* of lemon essential oil.

The greenness (a*) of fresh-cut kiwifruits is due to the presence of chlorophylls in mature green fruits [49]. About the a* value (Figure 1b), the HPMC-treated kiwi slices showed the lowest values during all the storage times compared to the other treatments, showing a greener color. The CTR and AVG showed a slight variation but not a significant difference (*p* ≤ 0.05) during cold storage. However, the LEO treatment had a different behavior compared to other treatments, probably due to the presence of lemon essential oil [27], which maintained a constant increase of this value, like the HPMC treatment, except after seven days of storage, which showed the same alterations of the CTR and AVG. The effect of lemon essential oil has also been described for strawberries coated with chitosan and oleic acid from Vargas et al. [50] and chitosan and lemon essential oil from Perdones et al. [42]. In particular, the results showed that the HPMC coating seemed to maintain the typical bright green color of kiwi slices, with a very little slight decrease. A similar trend has also been demonstrated by Chitravathi, K. et al. [51], which explained a delayed overall process in green chiles coated with shellac. Hence, due to the presence of the coatings, we have obtained a reduction of browning, as indicated by negative a* and high L* values. This behavior was confirmed by the results obtained for the browning index (BI), as shown in Figure 1c. In fact, during cold storage, the CTR and AVG treatments showed the same behaviors, exhibiting maximum BI. However, the lowest value was shown by the HPMC and LEO treatments, with the same trend during the storage period. In particular, starting from the seventh day, a peak of browning due to ripening processes was observed in all treatments. These differences were also visible in Figure 2, which shows the colors obtained from the total color difference (∆*E*) values in CIEL*a*b* converted to the RGB scale. This behavior could be associated with the presence of polysaccharides and antioxidant agents in the coatings, which decelerate the maturation processes, according to the other results obtained. In fact, membrane lesions allow the mixing of the polyphenol oxidase enzyme (PPO), normally separated, and oxidable substrates, leading to oxidation and browning [52].

### 3.2. Weight Loss and Firmness

**Figure 3:**
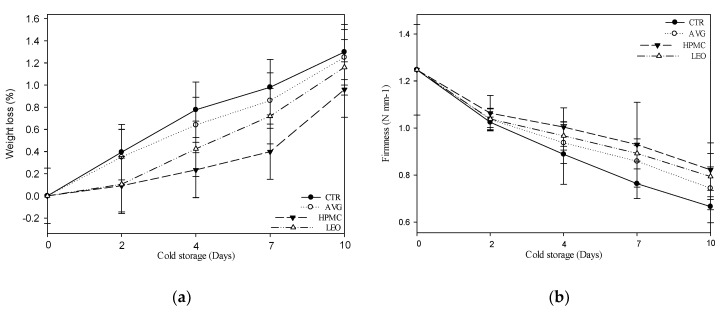
(**a**,**b**) Weight loss (%) and firmness (N·mm^−1^) of kiwifruit slices coated with AVG, HPMC and LEO treatments and not treated (CTR) at day 0 and after 2, 4, 7 and 10 days of cold storage at 4 ± 1 °C and 90% ± 5% relative humidity (RH) in dark conditions. Values indicate the means, and the bars indicate the standard deviations of the replicates (*n* = 3). Abbreviations: CTR, uncoated fruit slices, AVG, fruit slices coated with 40% *v/w* of *Aloe vera* gel only, HPMC, fruit slices coated with 40% *v/w* of *Aloe vera* gel + 0.1% *v/w* of hydroxypropyl methylcellulose and LEO, fruit slices coated with 40% *v/w* of *Aloe vera* gel + 0.1% *v/w* of lemon essential oil.

Weight loss is closely related to the firmness of the fruit, due to the consumers’ acceptability [53]. In Figure 3a, CTR and AVG-treated kiwi slices differed from the HPMC and LEO treatments due to a greater weight loss during the storage period. In fact, after ten days of storage, the untreated kiwi slices lost 1.30% of the weight, AVG 1.25%, HPMC 0.96% and LEO 1.16%.

Concerning firmness, the treatments showed (Figure 3b) the same trend during the 10 days of storage. They lost 40% of the firmness in samples treated with AVG, 34% in samples treated with HPMC and 36% in samples treated with LEO, while the untreated slices (CTR) lost 47% of the firmness during storage time. This decrease (both for treated and untreated kiwi slices) seemed to be due to the pectin degradation and the loss of the middle lamellae in the kiwifruits, which are processes that occur during the ripening [54].

The polysaccharide composition of *Aloe vera* gel [55] has proved to be highly effective as a moisture barrier. This behavior is due to its hygroscopic properties, which allowed the formation of a barrier to the diffusion of water between the fruit and environment, thus avoiding its external transfer [56]. Composite coatings of polysaccharides and lipids are known to increase the effectiveness of the water barrier with an increase in the lipid content, which, as a result, could further reduce the weight loss and firmness [57]. In fact, all the edible coatings seemed to reduce the water loss and inhibit the loss of firmness, but the *Aloe vera* gel alone was not sufficient to reduce gas exchanges with the surrounding environment. A similar result was obtained from Farina et al. [27] in fresh-cut apples.

In this context, the HPMC apparently inhibited both the weight loss and the pectin division along the cell wall, which was also confirmed by other studies on citrus fruits [57,58], apples [27] and plums [59], but also, the storage temperature had important effects on the coating performances.

In the case of the LEO treatment, the obtained results suggested that hydrophobic essential oils acted as barriers against the evaporation of moisture, which directly caused the loss of weight and firmness of the fruit, according to Choi et al. [59]. Finally, the presence of acids and antioxidant agents in the coating may have a better ability to adapt to changes in the surface of the fruit slices as they loses weight and firmness [32].

### 3.3. Total Soluble Solids Content, Titratable Acidity and pH

Concerning the SSC (Table 1), the HPMC and LEO treatments had slight increases during the first two days of storage in respect to the other treatments. The highest value of °Brix was obtained in the AVG treatment on the fourth day of storage (14.30), while all other treatments, including the CTR, had rather similar values. After that, they peaked on the seventh day of storage. All treatments showed a decrease in the soluble solids content during the last three days of storage, reaching values close to 14.00 °Brix, probably due to the initiation of degradation processes. In fact, the evolution of the soluble solids content demonstrates the natural ripening processes.

The TA decreased during storage time, but the HPMC and LEO showed a slower decrease compared to the CTR and AVG, which behaved almost the same way. As the fruit ripened, the amount of organic acids decreased, causing a sour taste, in accordance with the results obtained in sensory analysis.

In fact, during metabolic processes, organic acids are used as substrates in cellular respiration. For this reason, the TA is reduced, and the SSC is increased [60]. This ratio shows the ripening index of the fruit [42]. The coated samples containing LEO and HPMC showed lower SSC/TA ratios at the end of the storage (*p* < 0.05), indicating the possible positive effect of the essential oil agents on the metabolic activity of the fruits and the components of the polysaccharide matrix. Some authors have reported similar interactions [61] that may influence the metabolic pathway of the fruit and senescence.

These results are in agreement with the results obtained for the pH, which remains almost constant without significant differences (*p* ˂ 0.05) in all treatments (data not shown), according to other authors [25,62].

### 3.4. Microbiological Analysis

The loads of bacteria and fungi detected on the different samples collected during the experimentation are reported in Table 2 and Table 3, respectively. The microbiological analyses of the three edible coatings did not provide evidence of the presence of any of the microbial group objects of investigation. No colonies of pseudomonads, responsible to the alteration of different vegetables and fruits [63,64], were detected in any of the samples analyzed. The amount of Enterobacteriaceae, a family that includes several potential pathogenic microorganisms [65], were below the detection limit during the entire period of analysis. The same trend was reported by Allegra et al. [13] on kiwifruit slices coated with Opuntia Ficus-Indica mucilage. According to Tukey’s test, statistically significant differences were found for the levels of TMM, TPM, yeasts and molds between the control treatment and coated slices (AVG, HPMC and LEO). These differences were observed at four days of storage for TMM and yeast and at seven days of storage for TPM and molds and, in particular, when the AVG, HPMC and LEO treatments showed lower microbial cell densities than the control treatment. This trend is mainly due to the ability of *Aloe vera*-based edible coatings to reduce the microbial growth in fresh-cut fruits and vegetables [19,66]. However, the lowest levels of bacteria and fungi were detected for the trials HPMC and LEO that included the addition of hydroxypropyl methylcellulose or lemon essential oil, respectively. HPMC is a natural selective barrier to gas, moisture and solute migration and effectively reduces the microbial growth [67], while essential oils extracted from Citrus lemons are highly effective against food spoilage and/or pathogenic microorganisms [68,69]. However, the microbial spoilage in fresh-cut fruits is usually detected by consumers when aerobic bacteria and fungi reach levels above 7 and 5 Log CFU/g, respectively [70]. The concentration of these microorganisms in all productions increased during the entire period of observation, but the levels of the yeasts did not exceed 5 Log CFU/g. These results showed that the application of the three *Aloe vera* gel-based edible coatings, although not able to inhibit the microbial growth, limited significantly their development in coated fruits, prolonging their shelf lives.

### 3.5. Sensory Analysis

**Figure 4:**
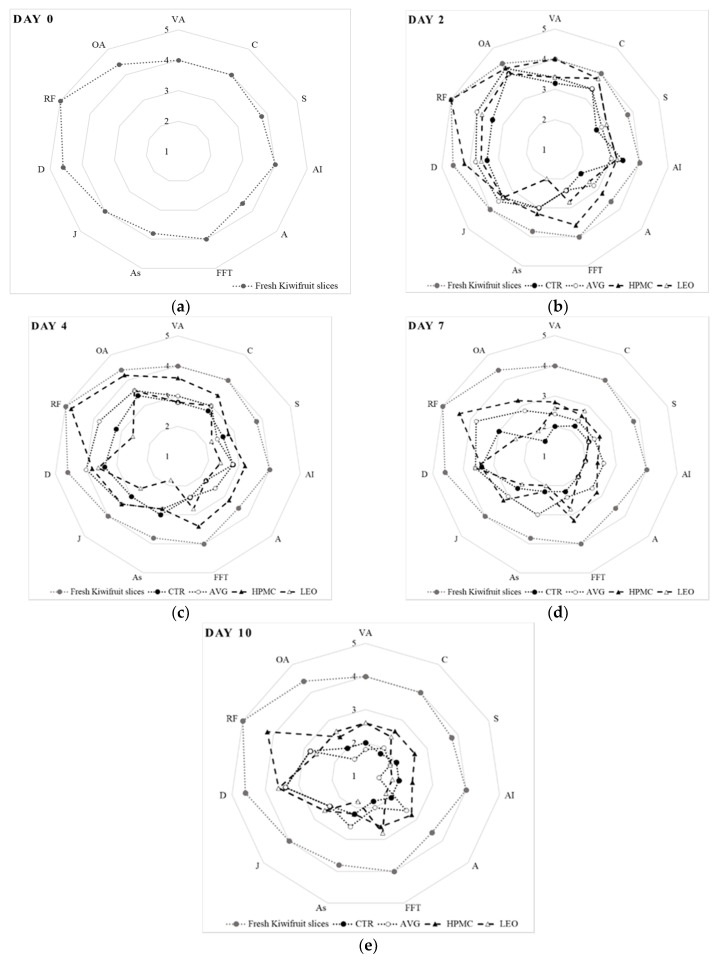
(**a**) Sensory evaluation of fresh kiwifruit slices at day 0. Descriptors of marketability: visual aspect (VA), color (C), sweetness (S), aroma intensity (AI), acidity (A) and fibrous flesh texture (FFT); descriptors of physiological alterations: astringency (As), juiciness (J), dehydration (D) and rare flavors (RF). Overall assessment (OA). For the marketability parameters, the judges assessed the descriptors as follows: 1 = extremely unacceptable, 2 = inacceptable, 3 = limit of marketability, 4 = good and 5 = excellent. For the physiological alteration parameters, the judges evaluated the descriptors as follows: 1 = extreme presence, 2 = important presence, 3 = acceptable, limit of consumption, 4 = light presence and 5 = absence. (**b**,**c**) Sensory evaluation of fresh-cut kiwifruit slices with and without edible coatings at day 2 and day 4, compared with fresh (day 0). Descriptors of marketability: visual aspect (VA), color (C), sweetness (S), aroma intensity (AI), acidity (A) and fibrous flesh texture (FFT); descriptors of physiological alterations: astringency (As), juiciness (J), dehydration (D) and rare flavors (RF). Overall assessment (OA). For the marketability parameters, the judges assessed the descriptors as follows: 1 = extremely unacceptable, 2 = inacceptable, 3 = limit of marketability, 4 = good and 5 = excellent. For the physiological alteration parameters, the judges evaluated the descriptors as follows: 1 = extreme presence, 2 = important presence, 3 = acceptable, limit of consumption, 4 = light presence and 5 = absence. Abbreviations: CTR, uncoated fruit slices, AVG, fruit slices coated with 40% *v/w* of *Aloe vera* gel only, HPMC, fruit slices coated with 40% *v/w* of *Aloe vera* gel + 0.1% *v/w* of hydroxypropyl methylcellulose and LEO, fruit slices coated with 40% *v/w* of *Aloe vera* gel + 0.1% *v/w* of lemon essential oil. (**d**,**e**) Sensory evaluation of fresh-cut kiwifruit slices with and without edible coatings at day 7 and day 10, compared with fresh (day 0). Descriptors of marketability: visual aspect (VA), color (C), sweetness (S), aroma intensity (AI), acidity (A) and fibrous flesh texture (FFT); descriptors of physiological alterations: astringency (As), juiciness (J), dehydration (D) and rare flavors (RF). Overall assessment (OA). For the marketability parameters, the judges assessed the descriptors as follows: 1 = extremely unacceptable, 2 = inacceptable, 3 = limit of marketability, 4 = good and 5 = excellent. For the physiological alteration parameters, the judges evaluated the descriptors as follows: 1 = extreme presence, 2 = important presence, 3 = acceptable, limit of consumption, 4 = light presence and 5 = absence. Abbreviations: CTR, uncoated fruit slices, AVG, fruit slices coated with 40% *v/w* of *Aloe vera* gel only, HPMC, fruit slices coated with 40% *v/w* of *Aloe vera* gel *+* 0.1% *v/w* of hydroxypropyl methylcellulose and LEO, fruit slices coated with 40% *v/w* of *Aloe vera* gel + 0.1% *v/w* of lemon essential oil.

Many times, consumers are viewing a product for the first time on the basis of its appearance, while factors such as taste and texture lead them to repeat the purchase [71]. Figure 4a–e shows the spider plots containing both the descriptors of marketability and descriptors of the physiological alterations. We have found large differences in the sensory attributes between treated and untreated kiwi slices. In the first day of the sensory analysis (Figure 4a), the kiwifruit slices were appreciated by consumers for all parameters. In particular, they did not detect any unusual flavor (RF) and gave an overall final assessment (OA) equal to 4.4, i.e., the *a*-value equivalent to a good or excellent degree of marketability.

From day two of the storage (Figure 4b), the kiwi slices started to show a decrease in visual appearance (VA) values. This behavior was probably due to an appearance that is no more intact and homogeneous, as on day 0. In particular, the CTR and AVG had values close to 2 at the end of the storage period, which indicated that the product was commercially unacceptable according to the judges. The highest values, on the other hand, were given to the HPMC and LEO, which maintained the appearance of the kiwi slices almost unchanged (2.6). The parameter that most deviated from the average values was the degree of astringency (As) for the LEO treatment (2), because already from the second day of storage, the judges detected the taste of lemon essential oil that covered the taste of kiwifruit, according to Perdones A. et al. [42], who found that the typical strawberry aroma and taste was masked by the chitosan and lemon essential oil coating.

The judges determined that HPMC treatment remained the most hydrated (D parameter) sample for the entire storage period (4.2), in accordance with the results obtained in the weight loss and firmness analysis. In fact, most of the undesirable changes in the fruit texture were caused by enzymes such as β-galactosidase and exo-polygalacturonase, which solubilized pectin in the cell walls [72]. Finally, as mentioned above, rare flavors (RF) were found in the CTR and LEO treatments. The final evaluation gave higher values in the HPMC and AVG (4.2).

On day four of storage (Figure 4c), the HPMC treatment tended to maintain the highest values for all evaluated parameters, except for the As, which was lower than the CTR (2.8 and 3, respectively). The lowest values, on the other hand, were highlighted in the LEO treatment for the S, As, J and RF parameters.

The CTR and AVG did not differ much, except for the RF, because an herbaceous taste was found in the slices treated with AVG, while in the CTR, a fibrous texture of the pulp was found, associated with the fibrous texture of canned pineapple, according to Marsh K. [47].

On day seven, all the values were at the limit of marketability (Figure 4d). In particular, the CTR had a visual aspect (VA) equal to 1, while the HPMC had all values still above average. The AVG showed a higher aroma intensity (AI) value (2.6) and a higher astringency (As) value (3). Finally, on the tenth and last day of storage (Figure 4e), all the parameters assumed equal values in terms of dehydration (D) and fibrous texture (FFT), which were higher in the HPMC and LEO and lower in the CTR and AVG, while the acidity (A) was higher only in the HPMC treatment.

The results obtained from the overall evaluation (OA) showed that the judges appreciated more the slices of kiwi treated with the HPMC (2.4) and LEO (2.6), as they maintained higher hydrations and textures, while the CTR and AVG were not appreciated for the formation of rare flavors and loss of textures and colors.

These results are in agreement with the studies carried out by Martínez-Romero et al. [66], which reported that *Aloe vera* gel gave a natural look attractive to the cherry, while Valverde et al. [19] reported that grapes treated with *Aloe vera* were better than untreated ones in terms of crunchiness, juiciness and acidity. Finally, O’Connor-Shaw et al. [72] confirmed the results obtained for the visual aspect and aroma intensity, which gradually decreased during cold storage, while Farina et al. [27] stated that, in slices of apples treated with the same edible coating, there were no rare flavors given by lemon essential oil or hydroxypropyl methylcellulose.

## 4. Conclusions

Our results, in accordance with some studies concerning the application of edible coatings, state that it is possible to prolong the marketability of minimally processed fruit by the application of chemical-free edible coatings. This study case proved the ability of edible coatings to maintain the quality characteristics of fresh-cut fruits. In particular, the HPMC and LEO treatments maintained the highest values in terms of firmness, brightness, greenness and soluble solid contents and lower values of weight loss, browning index and the SSC/TA ratio, as it acts as a barrier to gaseous exchanges. We also believed that the interaction of the HPMC and LEO with *Aloe vera* gel further reduced the microbial load, compared to the CTR and AVG.

The sensory analysis confirmed these results and claimed that the edible coating based on *Aloe vera* gel and the HPMC did not alter the natural taste of the kiwi slices, unlike the LEO treatment, which highlighted the characteristic taste of lemon essential oil, and the AVG treatment, which highlighted a herbaceous taste that was not perceived in the other treatments.

Finally, the AVG treatment showed few differences compared to the CTR treatment, so it can be said that *Aloe vera* gel improved the intrinsic characteristics of the kiwi slices, but when applied with a natural antioxidant and gelling agents (as lemon essential oil and hydroxypropyl methylcellulose), showed a greater ability to preserve the quality of the minimally processed fruit.

## Figures and Tables

**Table 1:** Evolution of the soluble solids content (SSC), titratable acidity (TA) and the SSC/TA ratio on day 0 (fresh), 2, 4, 7 and 10.

Treatments	0 d				2 d				4 d				7 d				10 d			
SSC (°Brix)																				
CTR	13.03	±	1.8	cA	13.7	±	1.89	bB	13.7	±	0.42	bB	14	±	0.81	aB	13.6	±	1.61	bB
AVG	13.03	±	1.8	cA	13.9	±	0.21	bA	14.3	±	4.08	aA	14.2	±	0.56	abA	13.8	±	1.76	bcA
HPMC	13.03	±	1.8	cA	13.1	±	0.62	abC	13.7	±	0.96	abB	14.1	±	0.31	aB	13.9	±	2.27	bA
LEO	13.03	±	1.8	cA	13.1	±	0.78	cC	13.3	±	1.86	bC	14.1	±	0.38	aB	13.9	±	0.87	aA
TA (g citric acid·L^−1^)																				
CTR	0.77	±	0.1	aA	0.44	±	0.25	bB	0.27	±	0.09	cB	0.23	±	0.12	cB	0.18	±	0.05	cC
AVG	0.77	±	0.1	aA	0.43	±	0.1	bB	0.37	±	0.07	bB	0.32	±	0.1	bB	0.26	±	0.14	bC
HPMC	0.77	±	0.1	aA	0.71	±	0.1	bAB	0.64	±	0.21	abA	0.6	±	0.16	abA	0.5	±	0.13	abA
LEO	0.77	±	0.1	aA	0.7	±	0.05	aA	0.53	±	0.13	bA	0.51	±	0.18	bA	0.42	±	0.13	bB
SSC/TA																				
CTR	17.00	±	0.7	bA	30.8	±	23.7	aA	50.2	±	16.4	abA	61.9	±	43.1	abA	77.2	±	36.8	abA
AVG	17.00	±	0.7	bA	32.4	±	8.72	abA	38.3	±	14.2	aAB	44.4	±	13.6	aA	53.9	±	54.7	abA
HPMC	17.00	±	0.7	aA	18.5	±	3.95	aBC	21.5	±	9.35	aB	23.4	±	5.64	aA	27.8	±	6.22	aA
LEO	17.00	±	0.7	bA	18.7	±	2.99	abC	25	±	9.99	abB	27.6	±	13.9	abA	32.8	±	12.9	aA

Data corresponds to the means ± standard deviation of three replicates. Means with different letters are significantly different at *p* ≤ 0.05 using Tukey’s test. Different capital letters denote significant differences (*p* ≤ 0.05) among different treatments for the same sampling time. Different lowercase letters denote significant differences (*p* < 0.05) among different sampling times for the same treatment. Abbreviations: CTR, uncoated fruit slices, AVG, fruit slices coated with 40% *v/w* of *Aloe vera* gel only, HPMC, fruit slices coated with 40% *v/w* of *Aloe vera* gel + 0.1% *v/w* of hydroxypropyl methylcellulose and LEO, fruit slices coated with 40% *v/w* of *Aloe vera* gel + 0.1% *v/w* of lemon essential oil.

**Table 2:** Total mesophilic and psychrotrophic bacterial loads of the fruit samples.

Treatments	TMM					TPM				
	0 d	2 d	4 d	7 d	10 d	0 d	2 d	4 d	7 d	10 d
CTR	<2a	<2a	3.39 ± 0.20a	3.99 ± 0.10a	4.73 ± 0.21a	<2a	<2a	<2a	4.07 ± 0.29a	4.88 ± 0.27b
AVG	<2a	<2a	2.67 ± 0.10b	3.47 ± 0.20b	4.08 ± 0.13b	<2a	<2a	<2a	3.35 ± 0.11b	4.01 ± 0.10b
HPMC	<2a	<2a	2.35 ± 0.19b	3.15 ± 0.09bc	3.65 ± 0.23b	<2a	<2a	<2a	2.94 ± 0.17b	3.78 ± 0.08b
LEO	<2a	<2a	2.37 ± 0.23b	2.97 ± 0.23c	3.65 ± 0.25b	<2a	<2a	<2a	3.00 ± 0.21b	3.73 ± 0.15b
*p*-value	NS	NS	0.0001	0.0001	0.001	NS	NS	NS	0.001	0.0001

Units are log CFU/g. Results indicate mean values ± S.D. of four plate counts (carried out in triplicate for two independent productions). Data within a column followed by the same letter are not significantly different according to Tukey’s test. Abbreviations: CTR, uncoated fruit slices, AVG, fruit slices coated with 40% *v/w* of *Aloe vera* gel only, HPMC, fruit slices coated with 40% *v/w* of *Aloe vera* gel + 0.1% *v/w* of hydroxypropyl methylcellulose, LEO, fruit slices coated with 40% *v/w* of *Aloe vera* gel + 0.1% *v/w* of lemon essential oil, TMM, total mesophilic microorganisms, TPM, total psychrotrophic microorganisms and NS, not significant (*p*-value ≥ 0.05).

**Table 3:** Yeast and mold loads of fruit samples.

Treatments	Yeast					Molds				
	0 d	2 d	4 d	7 d	10 d	0 d	2 d	4 d	7 d	10 d
CTR	<2a	<2a	3.21 ± 0.17a	3.64 ± 0.23a	3.97 ± 0.15a	<2a	<2a	<2a	3.88 ± 0.17a	4.46 ± 0.17a
AVG	<2a	<2a	2.55 ± 0.14b	2.87 ± 0.19b	3.37 ± 0.20b	<2a	<2a	<2a	3.13 ± 0.10b	3.71 ± 0.09b
HPMC	<2a	<2a	2.21 ± 0.25b	2.44 ± 0.27b	3.05 ± 0.25b	<2a	<2a	<2a	2.84 ± 0.25b	3.65 ± 0.21b
LEO	<2a	<2a	2.29 ± 0.29b	2.50 ± 0.23b	3.07 ± 0.10b	<2a	<2a	<2a	2.90 ± 0.21b	3.67 ± 0.20b
*p*-value	NS	NS	0.002	0.001	0.001	NS	NS	NS	0.001	0.001

Units are log CFU/g. Results indicate mean values ± S.D. of four plate counts (carried out in triplicate for two independent productions). Data within a column followed by the same letter are not significantly different according to Tukey’s test. Abbreviations: CTR, uncoated fruit slices, AVG, fruit slices coated with 40% *v/w* of *Aloe vera* gel only, HPMC, fruit slices coated with 40% *v/w* of *Aloe vera* gel + 0.1% *v/w* of hydroxypropyl methylcellulose, LEO, fruit slices coated with 40% *v/w* of *Aloe vera* gel + 0.1% *v/w* of lemon essential oil, TMM, total mesophilic microorganisms, TPM, total psychrotrophic microorganisms and NS, not significant (*p*-value ≥ 0.05).
